# Supplementary material for: In vitro role of Rad54 in Rad51-ssDNA filament-dependent homology search and synaptic complexes formation
Source: Nat Commun. 2019 Sep 6;10:4058. doi: 10.1038/s41467-019-12082-z (PMC6731316; doi:10.1038/s41467-019-12082-z)
Supplement: Supplementary file 1 — Supplementary Information [file 41467_2019_12082_MOESM1_ESM.pdf]

Supplementary information

In vitro role of Rad54 in Rad51-ssDNA filament dependent homology search and synaptic complexes formation, Moreira Tavares et al.

Name	sequence
Cy5-2574^{+}	5’-(Cy5)CGACGGTCAAGTCAGAGG
biotin-4014^{-}	5’-(Btn)GGATCTCAACAGCGGTAA
biotin-2574^{+}	5’-(Btn)CGACGGTCAAGTCAGAGG
3185^{-}	TTAACGTGAGTTTTCGTTCC

Supplementary Table 1: oligonucleotides used in the study

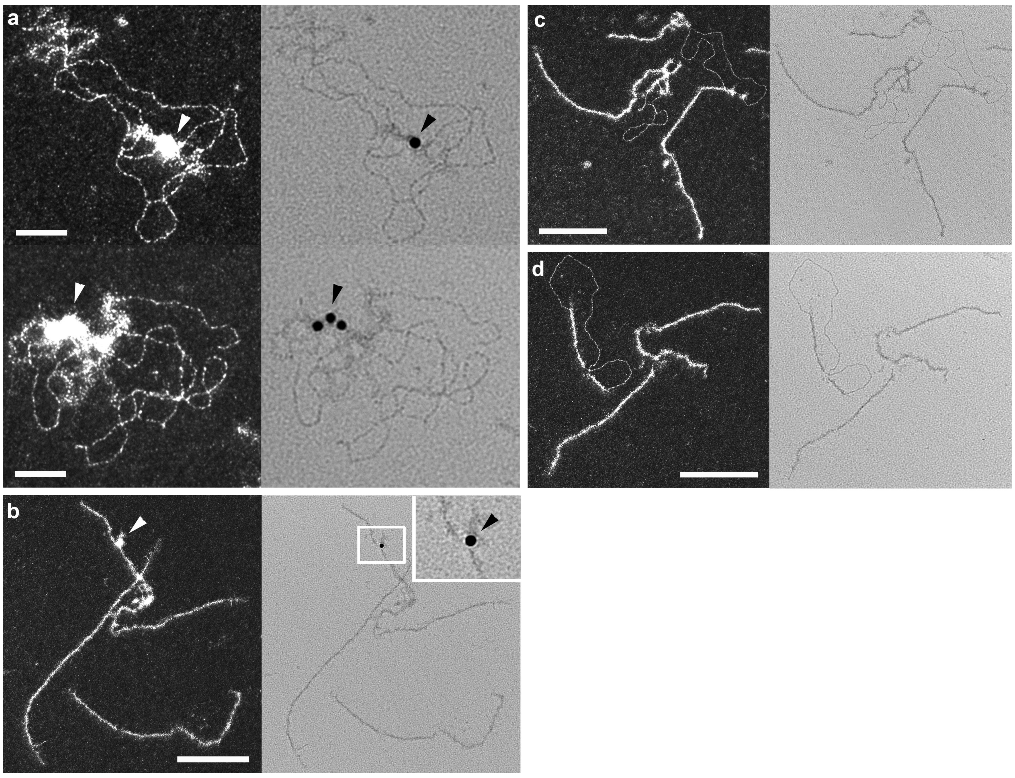

Supplementary Figure 1. EM representative views of Rad54 immunodetection during D-loop reaction: a) Rad54 is detected in the contact zone of joint molecules (scale bars: 50 nm); b) Rad54 is detected inside the Rad51 nucleofilment (scale bar: 200 nm); c) Control experiment using a primary antibody directed against yeast Srs2. No gold beads are detected demonstrating the specificity of the labeling (scale bar 200 nm); d) Reaction in absence of Rad54, no immunolabeling is shown (scale bar 200 nm). N = 2. Source data are provided as a Source Data file.

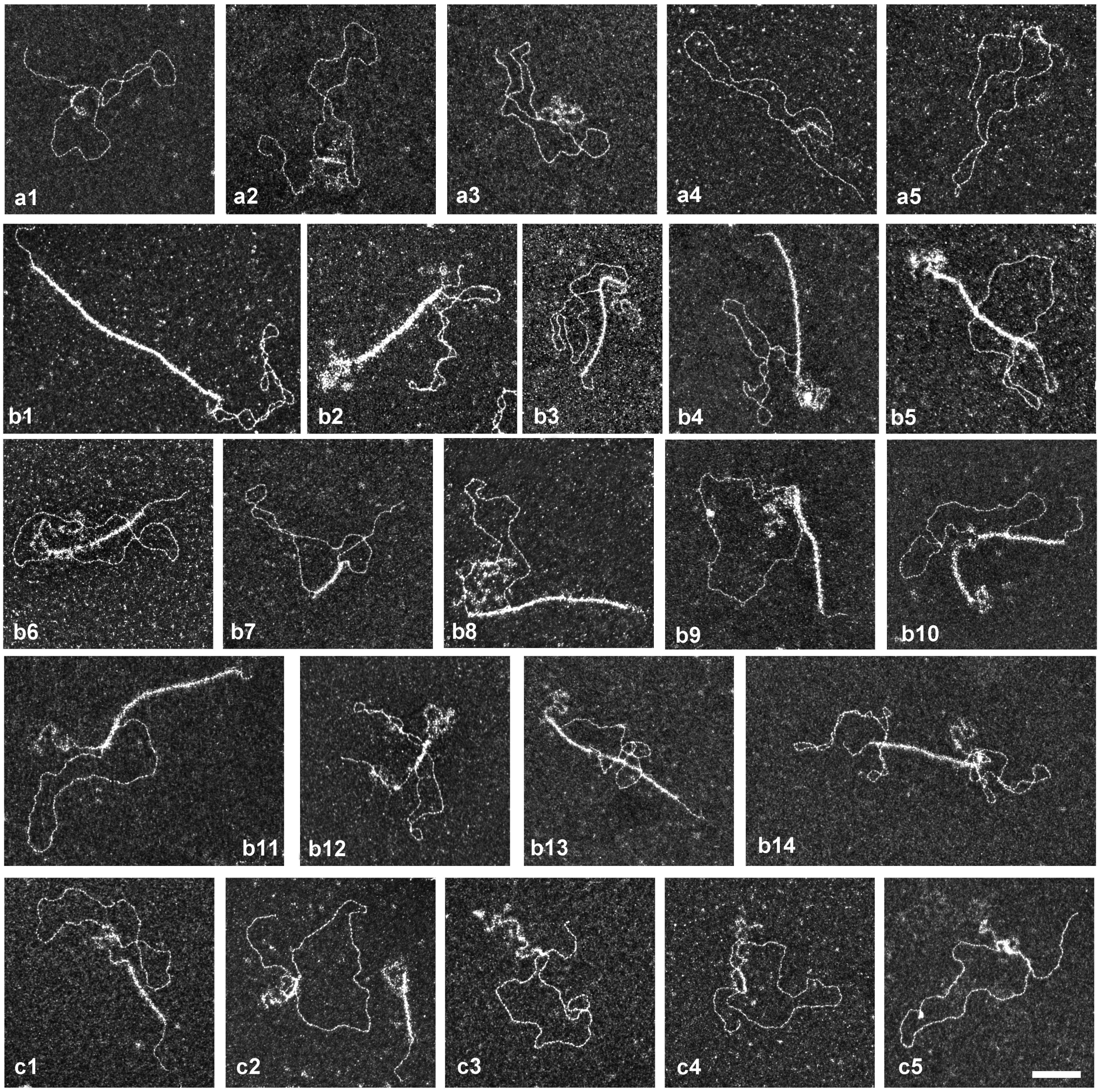

Supplementary Figure 2. Picture gallery of joint molecules generated during D-loop *in vitro* assay with Rad51 and Rad54 observed by EM: a) D-loops from deproteinized samples (see **Fig. 1**); b) synaptic complexes and c) D-loops from DNA-protein samples (see **Fig. 2**); and d) D-loops with visibly displaced strand. White bar represents 100 nm. Source data are provided as a Source Data file.

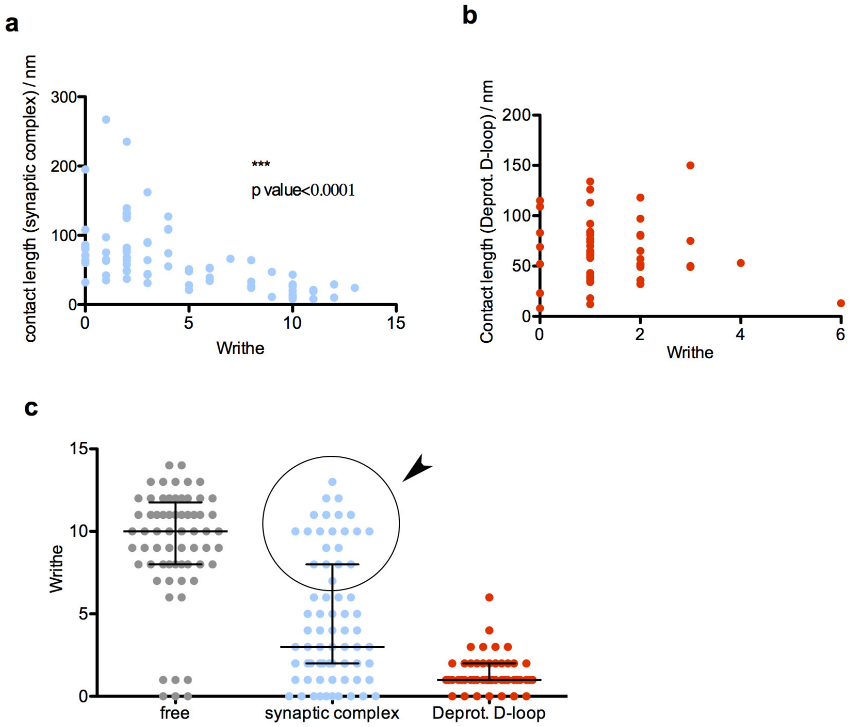

Supplementary Figure 3. (**a**) Relationship between synaptic complexes contact length and the absolute writhe (number of times the DNA double helix crosses itself). (**b**) Relationship between deproteinized D-loops contact length (heteroduplex) and the absolute writhe. (**c**) Distribution of the absolute writhe in the free DNA donor, synaptic complexes and deproteinized D-loops. Graphs show median with interquartile range, free donor (n=64), synaptic complexes (n=76), deproteinized D-loops (n=49), from three independent replicates. The round with the arrow points out a subpopulation of synaptic complex molecules without any change in the writhe number. For (a) and (b), a Spearman correlation test was applied, in which (a) shows a significant correlation (***), whereas (b) does not. Source data are provided as a Source Data file.
